# Linkage disequilibrium, SNP frequency change due to selection, and association mapping in popcorn chromosome regions containing QTLs for quality traits

**DOI:** 10.1590/1678-4685-GMB-2015-0126

**Published:** 2016

**Authors:** Geísa Pinheiro Paes, José Marcelo Soriano Viana, Fabyano Fonseca e Silva, Gabriel Borges Mundim

**Affiliations:** 1 Departamento de Biologia Geral, Universidade Federal de Viçosa, Viçosa, MG, Brazil; 2 Departamento de Zootecnia, Universidade Federal de Viçosa, Viçosa, MG, Brazil

**Keywords:** gametic phase disequilibrium, GWAS, candidate gene

## Abstract

The objectives of this study were to assess linkage disequilibrium (LD) and selection-induced changes in single nucleotide polymorphism (SNP) frequency, and to perform association mapping in popcorn chromosome regions containing quantitative trait loci (QTLs) for quality traits. Seven tropical and two temperate popcorn populations were genotyped for 96 SNPs chosen in chromosome regions containing QTLs for quality traits. The populations were phenotyped for expansion volume, 100-kernel weight, kernel sphericity, and kernel density. The LD statistics were the difference between the observed and expected haplotype frequencies (D), the proportion of D relative to the expected maximum value in the population, and the square of the correlation between the values of alleles at two loci. Association mapping was based on least squares and Bayesian approaches. In the tropical populations, D-values greater than 0.10 were observed for SNPs separated by 100-150 Mb, while most of the D-values in the temperate populations were less than 0.05. Selection for expansion volume indirectly led to increase in LD values, population differentiation, and significant changes in SNP frequency. Some associations were observed for expansion volume and the other quality traits. The candidate genes are involved with starch, storage protein, lipid, and cell wall polysaccharides synthesis.

## Introduction

Linkage disequilibrium (LD) or gametic phase disequilibrium is the difference between haplotype frequency products (P(AB).P(ab) – P(Ab).P(aB)) (Kempthorne, 1957). Because this difference corresponds to the covariance between values of alleles at two loci (Weir, 2008), LD is commonly defined as the non-random association of alleles at different loci. LD between molecular markers and genes, the basis of quantitative trait locus (QTL) mapping, association mapping, and genomic selection, is due to or affected by selection, mutation, population admixture, genetic drift, outcrossing, inbreeding, and recombination (Gupta*et al.*, 2005). With respect to biallelic markers, the most common statistics to measure LD in a population are the difference between the observed and expected (under linkage equilibrium) haplotype frequencies (D), the proportion of D relative to the expected maximum value in the population (D'), and the square of the correlation between the values of alleles at two loci (r^2^) (Flint-Garcia *et al.*, 2003).

Association mapping refers not only to the identification of QTLs, but also to the identification of candidate genes based on statistical significance between markers and phenotype. Its main advantages relative to QTL mapping are the use of breeding population instead of population derived by crossing two inbred or pure lines and more precise identification of candidate genes (Flint-Garcia *et al.*, 2005). However, association mapping is only capable of identifying effects of alleles present in reasonably high frequency in a population. In addition, the efficiency of association mapping is significantly influenced by relatedness and population structure, which can generate spurious associations, that is, associations between unlinked marker and QTL (Weir, 2010). The association mapping methodologies are the candidate gene approach and the genome-wide association study (GWAS) (Rafalski, 2010). Both methods have been successfully used to determine the genetic basis of important complex traits and to identify some of the key genes.

In maize (*Zea mays* L.), LD analyses and association studies have been performed using inbred line panels. The LD analysis performed by Van Inghelandt *et al.* (2011)was based on 1,537 inbreds genotyped for 359 simple sequence repeat (SSR) loci and 8,244 single nucleotide polymorphisms (SNPs). Considering only linked markers, LD under low (SSR) and high (SNP) marker densities was comparable for Flint and Lancaster heterotic pools. For Stiff Stalk Synthetic (SSS) and Iodent heterotic pools, the average LD based on SNPs was 45 to 52% greater than that based on SSR markers. Truntzler *et al.*(2012) assessed LD in a panel of 314 dent inbreds genotyped for 979 SNPs. They observed an r^2^ value of 0.20 for SNPs at a spacing of 200 kb. Based on a panel of 240 inbreds genotyped for 29,619 SNPs, Thirunavukkarasu *et al.* (2013) estimated r^2^values ranging from 0.21 to 0.25. LD blocks were observed on all chromosomes, with the LD decay occurring over regions of 200-300 kb.

Association mapping in maize has been effective for identifying candidate genes for complex traits such as pathogen resistance, root development, drought tolerance, chilling tolerance, oil biosynthesis, plant architecture, kernel composition, flowering, and metabolic processes. Using simulated and field data from five plant species including maize, Stich and Melchinger (2009) and Yang *et al.*(2010) compared association mapping methods. They concluded that a mixed-model approach using a kinship matrix to correct for relatedness was the best method. This approach outperformed a model controlling for relatedness and population structure because the spurious associations could not be completely controlled by population structure. Thirunavukkarasu*et al.* (2014) assessed 240 inbreds under water-stressed and well-watered environments. They measured anthesis-to-silking interval, grain yield, 100-kernel weight, and four ear traits, and carried out association mapping based on 29,619 high-quality SNPs. Fifty and 70 SNPs were strongly associated with tolerance to water stress under stressed and well-watered environments, respectively. Significant SNPs were identified mainly on chromosomes 5 and 3 under the water-stressed environment and on chromosomes 10, 1, and 7 under well-watered conditions. Thirty-one of the SNPs detected under water-stressed conditions were situated near drought-tolerance genes.

To our knowledge, little information is available on LD and SNP frequency changes due to selection in maize and special maize breeding populations, nor have QTLs been identified in such populations by association mapping. Thus, our objectives were to assess LD and SNP frequency changes due to selection, and to perform association mapping in popcorn (*Zea mays* L. ssp. *everta*(Sturtev.) Zhuk.) chromosome regions containing QTLs for quality traits.

## Materials and Methods

### Populations

The populations employed in this study were Viçosa, Viçosa cycles 1 (c1) and 4 (c4) (obtained from Viçosa after one and four half-sib selection cycles, respectively), Viçosa cycle 2 (c2) fsf (derived from Viçosa after two full-sib selection cycles), Viçosa S_4_ (generated from four inbred progeny selection cycles applied in the Viçosa population), Beija-Flor c1 and Beija-Flor c4 (obtained from Beija-Flor c1 after three half-sib selection cycles), and UFV MP-1 and UFV MP-2 (derived from hybrids P622 and P625, respectively, developed by the Agricultural Alumni Seed Improvement Association, Romney, IN, USA). The first seven populations, representing tropical germplasm, were cultivated during the 2012-2013 growing season in an experimental field at the Federal University of Viçosa (UFV), Minas Gerais, Brazil. The populations UFV MP-1 and UFV MP-2, representing temperate germplasm, were cultivated in 20-L pots in a greenhouse at UFV, in 2014. Leaf samples of 100-150 young plants were collected from each population for genotyping.

The populations derived from Viçosa and Beija-Flor c1 were obtained by progeny and plant-within-progeny selection for expansion volume. In trials of non-inbred progeny, 196 progeny were assessed using a 14 x 14 lattice design with two replications, at the UFV experimental station located in Coimbra, Minas Gerais. The 20 superior half-sib families were recombined using one male row to four female rows. In the recombination plots with the 20 superior full-sib families, at least one female per family was crossed with a male from another progeny, providing 380 full-sib families. In the half-sib progeny recombination plots, 196 plants were selected, providing the half-sib families for the next cycle. The 196 full-sib families for the next cycle were selected based on the expansion volume of the female parent.

The inbred progeny were assessed in an experimental field at UFV using an incomplete block design with replications only for the controls (commercial hybrids and populations). Each incomplete block consisted of 10 progeny and the controls. The trials included 344 S_1_ progeny, 309 S_2_progeny, 277 S_3_ progeny, and 268 S_4_ progeny. In each progeny, three to five plants were selfed. The progeny for the next cycle were obtained by selecting the best families and then the superior selfed plants. Populations Viçosa S_1_ to Viçosa S_4_ were obtained by recombining all assessed inbred progeny. The progeny tests and recombination plots were conducted during 1998-1999 to 2007-2008 growing seasons. To assess expansion volume, we used a hot air popcorn popper (1,200 W) or a 27-L microwave oven (900 W), and samples of 30 g per plot and 10 g per plant.

### Genotyping

DNA was extracted using KitWizard Genomic DNA Purification kit according to the manufacturer's protocol with modifications. A Qubit 2.0 fluorometer (Life Technologies, Carlsbad, CA, USA) and a NanoVue spectrophotometer (GE Healthcare BioSciences Corporation, Piscataway, NJ, USA) were used to assess DNA quantity and purity level, respectively. Individuals were genotyped from 50 ng/μL DNA samples using GoldenGate assays (Illumina, San Diego, CA, USA). Genotyping was performed on an Illumina BeadXpress. Individuals were genotyped for 96 SNPs located in chromosome regions containing QTLs for the following popcorn quality traits: expansion volume, flake volume, unpopped kernel number, and flake size (Table 1). The SNPs were selected from the maize 56-kb SNP50 array (56,110 SNPs from ~19,000 genes) on the basis of locations of the SSR primers flanking the QTLs mapped by Li *et al.* (2006, 2007, 2008, 2009), Babu *et al.* (2006), andLu *et al.* (2003) and by using information in Maize Genetics and Genomics (MaizeGDB) and National Center for Biotechnology Information (NCBI) databases. Two SNPs did not map to any assembly. The number of genotyped plants ranged from 38 to 113. Genotypes were assigned using Illumina GenomeStudio (version 2011.1), with the GC score specified as 0.25. The average distance between adjacent SNPs was 9.1 Mb, and within bins, 464 kb.

**Table 1:** Name and location of the true and simulated SNPs.

Name	Chr.	Position (bp)	Bin	Name	Chr.	Position (bp)
PUT-163a-91054912-4739	-	-	-	-	-	-
PUT-163a-16922676-1070	-	-	-	-	-	-
SYN6001	1	2208377	1.01	1	1	4464052
SYN27251	1	2432669	1.01	2	1	4918980
PUT-163a-5499487-2275	1	2536526	1.01	3	1	6747184
SYN38927	1	2691547	1.01	4	1	6822691
SYN6413	1	7961412	1.01	5	1	7271821
PZE-101014003	1	7993526	1.01	6	1	8098952
PZE-101014266	1	8095694	1.01	7	1	8662055
SYN11901	1	8510819	1.01	8	1	9201172
SYN11909	1	8512237	1.01	9	1	10247073
PZA-000175002	1	8553473	1.01	10	1	12041260
SYN20196	1	15512478	1.02	11	1	14985200
PUT-163a-16922676-1073	1	46070067	1.03	12	1	44959476
SYN11221	1	66980686	1.04	13	1	67180473
SYN11222	1	66981108	1.04	14	1	68864563
SYN17701	1	67062198	1.04	15	1	68909790
PZE-101083826	1	72067065	1.04	16	1	69218117
SYN38509	1	72105795	1.04	17	1	70928139
SYN38510	1	72105859	1.04	18	1	72494781
PZE-101120556	1	148525047	1.05	19	1	146847931
PZE-101120639	1	148566496	1.05	20	1	148129135
PZE-101120645	1	148566793	1.05	21	1	149660797
PZE-101131103	1	168256682	1.05	22	1	167602997
PZE-101131114	1	168257319	1.05	23	1	167714661
PZE-101131166	1	168429768	1.05	24	1	168981018
PZE-101162300	1	205544018	1.07	25	1	204597382
SYN422	1	236291257	1.08	26	1	234385544
SYN423	1	236296165	1.08	27	1	236155502
ZM011097-0676	1	236296417	1.08	28	1	236592209
SYNGENTA11666	2	205583015	2.08	29	2	0
PZE-102158721	2	206039441	2.08	30	2	855530
PUT-163a-148951348-515	3	5789819	3.02	31	3	5524536
SYNGENTA17024	3	5854017	3.02	32	3	5754583
PZE-103010658	3	5854416	3.02	33	3	7669980
SYN33444	3	15137742	3.04	34	3	14800309
SYN33443	3	15222626	3.04	35	3	15194865
SYN33442	3	15222864	3.04	36	3	16334091
PZE-103160210	3	211405876	3.08	37	3	209104813
PZE-103160218	3	211408703	3.08	38	3	210855530
PZE-103160227	3	211410592	3.08	39	3	211377457
SYN33394	3	215456783	3.08	40	3	213475311
PZE-103165953	3	215462316	3.08	41	3	215009460
PUT-163a-149100944-925	3	215513343	3.08	42	3	216145401
PZE-104008299	4	5595386	4.02	43	4	0
PZE-104033459	4	41873008	4.05	44	4	34923515
PZE-104033791	4	42284265	4.05	45	4	36303978
PZE-104033817	4	42288134	4.05	46	4	38019829
PZE-104033826	4	42306869	4.05	47	4	39186188
SYN22745	4	154716125	4.06	48	4	148089966
PZE-104080384	4	154716758	4.06	49	4	148380829
SYN509	5	4219552	5.01	50	5	0
SYN524	5	4254273	5.01	51	5	1575760
SYN526	5	4254700	5.01	52	5	1580048
PZE-105018859	5	8560599	5.02	53	5	4536728
SYN4651	6	147905068	6.05	54	6	146819366
SYN4646	6	147909387	6.05	55	6	148468338
SYN4642	6	147909507	6.05	56	6	148928894
PZE-106100715	6	153502018	6.05	57	6	151316528
PZE-106100720	6	153502756	6.05	58	6	152790375
PZE-106100728	6	153508407	6.05	59	6	153651642
PZE-106115889	6	161854167	6.07	60	6	159940903
PZE-106116148	6	161924948	6.07	61	6	161828934
PZE-106116156	6	161990361	6.07	62	6	162886520
SYN12692	6	164180276	6.07	63	6	164783844
PZA02688.2	6	164183687	6.07	64	6	166620102
SYN12698	6	164186726	6.07	65	6	167632980
PZE-107083430	7	125723001	7.02	66	7	0
PZE-107083429	7	125723685	7.02	67	7	1462112
PZE-107105783	7	157912189	7.04	68	7	14959671
SYN36108	7	157914433	7.04	69	7	16157837
PZE-107105855	7	157934803	7.04	70	7	17505219
PZE-108004863	8	4967925	8.01	71	8	0
PZE-108004875	8	4969812	8.01	72	8	1878784
PZE-108004908	8	5025189	8.01	73	8	2059971
PZE-108052599	8	92730672	8.03	74	8	86063553
PZE-108052600	8	92730702	8.03	75	8	87675003
PZE-108052603	8	92732462	8.03	76	8	87804291
PZE-108134983	8	173931575	8.09	77	8	166943466
SYN20808	8	173953651	8.09	78	8	167344955
SYN20806	8	173973257	8.09	79	8	168464798
PZE-108135203	8	174257043	8.09	80	8	170052048
SYN17231	10	4990421	10.01	81	9	1098717
SYN17233	10	4992947	10.01	82	9	2942947
PZE-110006423	10	4993061	10.01	83	9	4581985
PZE-110007091	10	5485252	10.02	84	9	5060295
PZE-110007194	10	5537112	10.02	85	9	6864235
SYN16757	10	5592617	10.02	86	9	7174065
PZE-110012640	10	11088851	10.02	87	9	9114563
PZE-110012671	10	11184622	10.02	88	9	10769264
PZE-110012682	10	11195007	10.02	89	9	12219177
PZE-110045755	10	86439624	10.03	90	9	85800064
SYN37480	10	86528026	10.03	91	9	87218643
SYN16982	10	114875299	10.04	92	9	113242081
SYN16979	10	114876217	10.04	93	9	114440567
PZE-110060686	10	114892724	10.04	94	9	116157425

### Phenotyping

Expansion volume was assessed in a 27-L microwave oven (900 W) using samples of 10 or 30 g per plant. To provide an estimate of error variance for expansion volume, two measurements were obtained for most plants in the temperate populations. Hundred-kernel weight was measured with an electronic scale. Average kernel sphericity was calculated as the ratio of geometric mean diameter (cubic root of the multiplied length, width, and depth) to kernel length (as measured with a digital caliper [0.005-mm precision]) of 10 randomly-selected kernels per plant (Tian *et al.*, 2001). To determine kernel density, 50 kernels were weighted and placed in a 100-mL beaker (1.0-mL precision) containing 50 mL of 90% aqueous ethanol. Kernel volume was obtained by subtracting 50 mL from the final volume (Vyn and Tollenaar, 1998). The number of phenotyped plants ranged from 43 to 108.

### Data simulation

Because no reference was available for interpreting the LD analysis results for the popcorn populations, we also analyzed two simulated populations. Simulated population 1 (Pop1) was a second generation composite obtained by crossing two populations in linkage equilibrium. This population was in LD only for linked markers and/or genes. The second simulated population (Pop2) was obtained from Pop1 after 10 cycles of random crosses assuming sample sizes of 100 and 300. The effective population sizes were 200 and 600, respectively. The program used for simulating genotypes and phenotypes - REALbreeding - has been developed by the second author using REALbasic software (Viana*et al.*, 2013). In the simulation process, we tried to reproduce the same distribution of SNPs observed in the popcorn populations. We simulated 1,170 SNPs on nine chromosomes, of which 94 were selected and analyzed (Table 1). The average distance between adjacent SNPs was 9.1 Mb.

Nineteen QTLs (candidate genes) and 81 minor genes affecting the expansion volume trait were randomly distributed along the nine chromosomes. Based on user input, which included minimum and maximum genotypic values for homozygotes, degree of dominance (d/a), direction of dominance, and broad sense heritability, the REALbreeding program provided the phenotypic values of each genotyped individual. The phenotypic values were computed from the true population mean, additive and dominance values, and error effects sampled from a normal distribution. The error variance was computed from the broad sense heritability. The minimum and maximum genotypic values of homozygotes were 5 and 50 mL g^-1^. We also defined bidirectional dominance (-1.2 ≤ (d/a)_i_ ≤ 1.2) and used a heritability of 50%. The proportion of the phenotypic variance explained by each QTL was set to 2.4%.

### Statistical analyses

Missing genotypes were imputed with Beagle 3.3.2 (Browning and Browning, 2007). PowerMarker 3.25 (Liu and Muse, 2005) was used to compute SNP frequencies, gene diversity (expected heterozygosity), and LD statistics and to perform Hardy-Weinberg equilibrium tests and association mapping based on analysis of variance (equivalent to a least-squares regression analysis). The fixation index (F_ST_) was computed using GenAlEx 6.5 (Peakall and Smouse, 2006). For the population structure analysis, we used the Structure software (Falush *et al.*, 2003). SAS (SAS Institute, 2007) was used to compare population means and to compute phenotypic correlations. We used the R packages MCMCpack (Martin *et al.*, 2011) and boa (Smith, 2007) for a Bayesian GWAS.

A SNP was considered to be non-polymorphic when the minor allele frequency (maf) was less than 1%. Only SNPs in Hardy-Weinberg equilibrium, as assessed using a chi-square test at the 5% significance level, were used for the LD analysis. The LD measures were D, D', and r^2^. The significance of a SNP frequency change was based on Waples (1989)assuming a 0.05% level of significance. For the population structure analysis, the burn-in period and the number of Markov chain Monte Carlo (MCMC) replications consisted of 5,000 and 25,000 iterations, respectively, and the number of assumed populations (K) was varied from 2 to 10. We ran the analysis under the no-admixture model with correlated frequencies. The most probable Kvalue was determined based on the inferred plateau method (Viana *et al.*, 2013). The least-squares association mapping used a Benjamini-Hochberg false discovery rate (FDR) of 5% (Benjamini and Hochberg, 1995). For the Bayesian GWAS, the burn-in period, number of MCMC replications, and sampling interval were 50,000, 100,000, and five, respectively. Significant SNP effects were identified using 95% highest posterior density (HPD) intervals.

### Candidate gene analysis

SNP sequences in FASTA format were obtained from the NCBI Database of Single Nucleotide Polymorphisms and used to perform BLAST searches against the 'B73'RefGen_v2 reference genome at the MaizeGDB. Information on gene products, expression, and ontology (biological process, molecular function, and cellular component) was obtained using the MaizeCyc database, the Maize eFP browser, and the Gramene database. To identify candidate genes, we searched up to 1 Mb upstream and downstream of each SNP region.

## Results

### LD analysis

**Figure 1:**
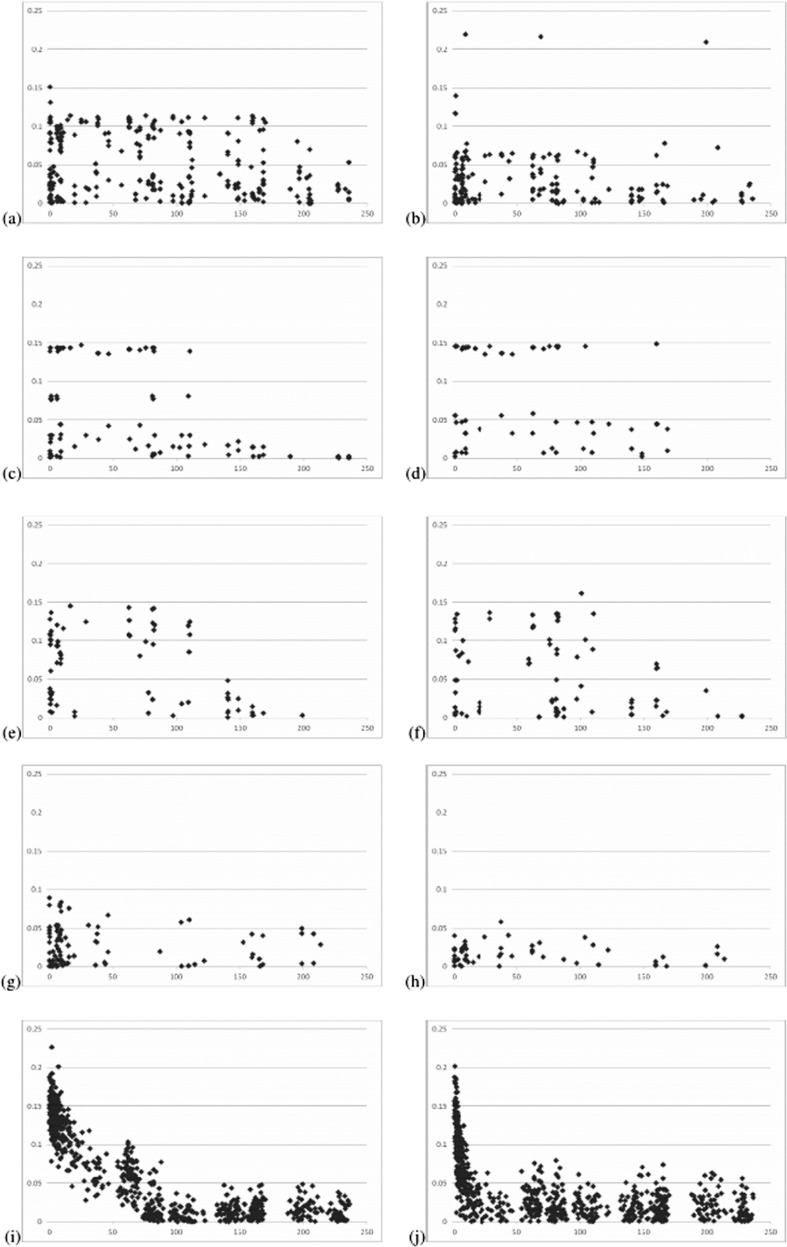
Relationship between the absolute D-value and distance (Mb) in the populations Viçosa (a), Viçosa c1 (b), Viçosa c4 (c), Viçosa c2 fsf (d), Viçosa S4 (e), Beija-Flor c1 (f), UFV MP-1(g), UFV MP-2 (h), Pop1, sample size 100 (i), and Pop2, sample size 100 (j).

The percentage of polymorphic SNPs in the popcorn populations ranged from 56.0 in Beija-Flor c4 to 93.0 in Viçosa c1 (Table 2), but the number of SNPs in Hardy-Weinberg equilibrium was the factor that negatively affected the LD analysis. The percentage of polymorphic SNPs in Hardy-Weinberg equilibrium ranged from 18.5 in Beija-Flor c4 to 65.5 in Viçosa. Expected heterozygosity ranged from 0.29 in UFV MP-1 to 0.39 in Viçosa c2 fsf. The minimum and maximum average D and r^2^ values were observed in Beija-Flor c4 and Viçosa c2 fsf, respectively. The lowest and highest average D' values were observed in UFV MP-2 and Viçosa c2 fsf, respectively. In the simulated populations, the number of polymorphic SNPs agreed with the value expected at the 5% significance level (at least 91), the expected heterozygosity approached the maximum value, and 10 generations of random mating decreased LD values. Also as expected, average LD values for linked SNPs were greater than those for linked and unlinked SNPs. This decrease occurred only in 50% of the cases for the popcorn populations. In the tropical popcorn populations, D-values greater than 0.10 were observed for SNPs separated by 100-150 Mb. Most of the D-values relative to the temperate populations were less than 0.05 (Figure 1). For the simulated populations, SNPs separated by more than 50 Mb generally exhibited a D-value less than 0.05, and SNPs separated by less than 10 Mb generally showed a D-value greater than 0.10.

**Table 2:** Population, number of genotyped individuals (Ng), number of polymorphic SNPs (Np), number of SNPs in Hardy-Weinberg equilibrium (Ne), average expected heterozygosity (He), and average absolute values of the LD measures by chromosome and for all SNPs^1^.

Population	Ng	Np	Ne	He	D	D'	r^2^	D^1^	D'^1^	r^21^
Viçosa	99	87	57	0.3049	0.0491	0.8059	0.1949	0.0401	0.7742	0.1660
Viçosa c1	73	89	44	0.3063	0.0298	0.7455	0.1461	0.0282	0.7624	0.1507
Viçosa c4	112	79	28	0.3739	0.0540	0.9107	0.3190	0.0650	0.9325	0.4008
Viçosa c2 fsf	113	76	24	0.3910	0.0741	0.9712	0.4128	0.0800	0.9842	0.4764
Viçosa S_4_	112	78	30	0.3833	0.0646	0.8014	0.3362	0.0666	0.7484	0.3552
Beija-Flor c1	107	82	31	0.3750	0.0520	0.8035	0.2593	0.0501	0.8134	0.2552
Beija-Flor c4	38	54	10	0.4300	0.0076	0.8108	0.0176	0.0061	0.7614	0.0207
UFV MP-1	95	66	37	0.2879	0.0268	0.7913	0.2005	0.0275	0.7732	0.1992
UFV MP-2	95	61	31	0.3141	0.0169	0.6975	0.1278	0.0165	0.6765	0.1312
Pop1	100	96	93	0.4869	0.0670	0.3392	0.1311	0.0272	0.1389	0.0296
Pop1	300	96	91	0.4858	0.0647	0.3233	0.1268	0.0195	0.0979	0.0232
Pop2	100	96	94	0.4792	0.0454	0.2450	0.0682	0.0252	0.1373	0.0214
Pop2	300	96	95	0.4774	0.0396	0.2107	0.0614	0.0174	0.0943	0.0140

### Efficiency of selection

According to a *t*-test at the 5% significance level, the selection process used on non-inbred and inbred progeny caused, with one exception, an increase in the mean expansion volume of the base populations (Viçosa and Beija-Flor c1) and an indirect decrease in 100-kernel weight (Table 3). Compared with tropical populations, temperate populations had a greater expansion volume and a lower 100-kernel weight, with lower phenotypic variance for both traits. The tropical and temperate populations had equivalent kernel sphericities and densities. The simulated populations showed the same mean and phenotypic variance regardless of sample size. Estimates of phenotypic correlations for expansion volume and kernel traits included some significant values (*p* < 0.05,*t*-test), but were characterized by intermediate (0.4) to low (0.2) magnitudes, especially for tropical populations. The sign of the estimates was also variable depending on the population.

**Table 3:** Population, number of phenotyped individuals (N), and minimum, average, maximum and variance for expansion volume (mL/g), 100-kernel weight (g), kernel sphericity, and kernel density (g/mL)

Population	N	Expansion volume				100-kernel weight				Kernel sphericity				Kernel density			
		Min.	Av.	Max.	Var.	Min.	Av.	Max.	Var.	Min.	Av.	Max.	Var.	Min.	Av.	Max.	Var.
Viçosa	93	9.0	26.9	50.0	48.33	15.5	21.3	28.8	9.14	0.60	0.75	0.93	0.003	0.89	1.33	1.63	0.012
Viçosa c1	43	18.0	30.9^1^	47.0	53.76	9.9	18.4^1^	25.1	12.61	0.65	0.79	0.96	0.007	0.68	1.29	1.62	0.040
Viçosa c4	108	15.0	31.0^1^	50.0	51.87	11.4	18.7^1^	28.7	9.80	0.62	0.75	0.92	0.003	0.92	1.32	1.95	0.023
Viçosa c2 fsf	89	10.0	30.5^1^	48.0	65.17	13.4	19.2^1^	26.2	8.99	0.63	0.74	0.93	0.004	1.01	1.32	1.91	0.020
Viçosa S_4_	98	15.0	28.6	44.0	43.44	11.6	19.7^1^	28.9	9.29	0.63	0.74	0.96	0.004	1.04	1.32	2.02	0.023
Beija-Flor c1	91	6.0	26.7	45.0	68.39	11.6	20.0	30.3	14.42	0.61	0.73	0.90	0.003	1.05	1.34	2.13	0.022
Beija-Flor c4	103	10.0	31.3^2^	51.0	61.70	10.1	18.1^2^	30.0	9.33	0.61	0.72	0.85	0.003	0.97	1.34	1.99	0.029
UFV MP-1	97	23.4	41.1	50.0	26.05	6.1	15.1	19.2	5.91	0.62	0.74	0.86	0.001	1.04	1.40	2.31	0.038
UFV MP-2	89	20.0	33.7	49.3	24.67	8.4	12.7	17.8	2.93	0.70	0.78	0.94	0.002	1.00	1.26	1.82	0.021
Pop1	100	22.9	33.4	43.2	16.84	-	-	-	-	-	-	-	-	-	-	-	-
Pop1	300	22.9	33.7	44.8	17.02	-	-	-	-	-	-	-	-	-	-	-	-
Pop2	100	24.5	33.6	43.1	17.23	-	-	-	-	-	-	-	-	-	-	-	-
Pop2	300	23.6	33.6	45.0	15.50	-	-	-	-	-	-	-	-	-	-	-	-

1 Significant at the 5% level by the *t*-test in relation to Viçosa;

2 significant at the 5% level by the *t*-test in relation to Beija-Flor c1.

### SNP frequency change

Selection for expansion volume was accompanied by increases in LD values in the Viçosa population (Table 2), population differentiation, and significant (non-random) changes in SNP frequency (Table 4). Increases in average LD values occurred only after four cycles of half-sib and inbred progeny selection and after two cycles of full-sib selection. With respect to linked SNPs, increases in average D, D', and r^2^ ranged from approximately 10% to 51%, 13% to 20%, and 64% to 118%, respectively. The increments for linked and unlinked SNPs were even higher. The genetic differentiation was proportional to the number of cycles. Relative to the Viçosa population, F_ST_ ranged from 0.09 in Viçosa c1 to 0.16 in Viçosa S_4_. The highest F_ST_ estimates, ranging from 0.19 to 0.34, were observed between tropical and temperate populations. The lowest value (0.00) was evidence of no genetic differentiation between Beija-Flor c1 and Beija-Flor c4 populations. Interestingly, genetic differentiation between the improved Viçosa and Beija-Flor populations was negligible (less than 0.05).

**Table 4:** Number of SNPs with significant allele frequency change by the Waples's test at 0.05% (N), and minimum, average, and maximum of the absolute value of the significant allele frequency changes in relation to population Viçosa, Beija-Flor c1, or Pop1

Population	N	Minimum	Average	Maximum
Viçosa c1	23	0.1848	0.2748	0.5493
Viçosa c4	41	0.0657	0.3004	0.8254
Viçosa c2 fsf	40	0.0657	0.2862	0.6537
Viçosa S_4_	35	0.1012	0.2999	0.7853
Beija-Flor c4	25	0.1635	0.2136	0.4299
Pop2^1^	0	0.0000	0.0551	0.1600
Pop2^2^	5	0.1017	0.1220	0.1433

1 Sample size 100;

2 sample size 300.

These findings are partially consistent with results from the population structure analysis. The inferred plateau method uncovered six subpopulations corresponding to UFV MP-1, UFV MP-2, Viçosa, three Viçosa-derived populations as a fourth subpopulation, Viçosa S_4_ and BeijaFlor derived populations as a fifth subpopulation, and a non-existent population (with individuals in the five previous subpopulations). Based on the Waples' test, the number of SNPs with significant (*p* < 0.05%) allele frequency changes relative to the base populations (Viçosa and Beija-Flor c1) ranged from 23 in Viçosa c1 to 41 in Viçosa c4, proportional to the number of cycles. The average change in SNP frequency ranged from 0.21 in Beija-Flor c4 to 0.30 in Viçosa c4 and Viçosa S_4_, which was also proportional to the number of cycles. Unexpected significant changes in SNP frequencies in the simulated population Pop2, with a sample size of 300, ranged from 0.10 to 0.14 (average of 0.12). It should be noted that one to seven SNPs in almost all bins showed significant frequency changes.

### Association mapping

Not a single significant association at a FDR of 5% was observed in the popcorn populations. Assuming a FDR of 10%, we found three associations for expansion volume, two associations for 100-kernel weight, and seven associations for kernel density in distinct populations (Table 5). The Bayesian GWAS uncovered no significant associations. With respect to the simulated populations, association mapping at 5% level of significance revealed 13 significant associations in Pop1 with a sample size of 300, five significant associations in Pop2 with a sample size of 300, no significant associations in Pop1 with 100 individuals, and, surprisingly, six significant associations in Pop2 with 100 individuals (Table 6). Most of the significant associations were uncovered by Bayesian GWAS. Analyses of both field and simulated data evidenced differences between least squares regression and Bayesian GWAS results, and between SNPs with significant associations. Only SNPs 30 and 87 showed an association in Pop2 at both sample sizes, identifying QTLs 5 and 18, respectively. These two QTLs were also identified from the analysis of Pop1 data with a sample size of 300, but the associations were with SNPs 31 and 84. Importantly, no false positives were apparent, and in 70% of the significant associations, the distance between the SNP and the candidate gene ranged from 121 to 11,867 kb (average of 4,117 kb).

**Table 5:** Location of SNPs with significant association at a false discovery rate of 10% for expansion volume, 100-kernel weight, or kernel density, in popcorn populations, and the candidate genes.

Trait	Population	Chr.	Position (bp)	SNP	Candidate gene
Expansion volume	Viçosa S4	1	168429768	PZE-101131166	GRMZM2G018472
		6	147905068	SYN4651	GRMZM2G058472
		8	173931575	PZE-108134983	GRMZM2G118462
100-kernel weight	Beija-Flor c4	1	168256682	PZE-101131103	GRMZM2G018472
	Viçosa c4	7	125723685	PZE-107083429	GRMZM2G133613
Kernel density	Viçosa c4	1	2208377	SYN6001	GRMZM2G109725
		3	15222626	SYN33443	GRMZM2G334628
		4	41873008	PZE-104033459	GRMZM2G138060
		8	173973257	SYN20806	GRMZM2G118462
	UFV MP-1	1	8510819	SYN11901	GRMZM2G009014
		10	11184622	PZE-110012671	GRMZM2G392513
		10	114892724	PZE-110060686	GRMZM2G049681

**Table 6:** Location of SNPs with significant association for expansion volume, based on a false discovery rate (FDR) of 5% or the 95% highest probability density (HPD) interval of the regression coefficients, and location of the closest QTL (candidate gene), in two simulated populations

Pop.	Sample	Chr.	Position (bp)	SNP	QTL	FDR	HPD int.
Pop1	300	1	8662055	7	-	ns	-2.89; −1.00
		1	12041260	10	-	0.0472	ns
		1	69218117	17	-	0.0409	ns
		1	70928139	18	-	0.0158	ns
		1	76126122	-	1	-	-
		1	146847931	20	-	0.0195	ns
		1	167714661	24	-	ns	-2.16; −0.38
		1	168860031	-	3	-	-
		1	168981018	25	-	0.0435	ns
		1	236592209	29	-	0.0150	ns
		1	245744736	-	4	-	-
		2	326508	-	5	-	-
		2	855530	31	-	ns	0.01; 1.54
		7	1462112	68	-	0.0247	ns
		7	4291016	-	14	-	-
		8	0	72	-	ns	-2.36; −0.29
		8	163719894	-	15	-	-
		9	4581985	84	-	ns	0.25; 1.89
		9	11190208	-	18	-	-
		9	58272812	-	19	-	-
		9	85800064	91	-	ns	0.03; 1.80
Pop2	300	2	0	30	-	ns	0.15; 1.74
		2	326508	-	5	-	-
		4	138458023	-	10	-	-
		4	148089966	49	-	ns	-1.67; −0.01
		6	133876175	-	13	-	-
		6	152790375	59	-	ns	-1.83; −0.03
		6	159940903	61	-	0.0049	ns
		9	7174065	87	-	ns	0.19; 2.27
		9	11190208	-	18	-	-
Pop2	100	1	168860031	-	3	-	-
		1	204597382	26	-	ns	-2.91; −0.24
		2	0	30	-	ns	0.41; 3.37
		2	326508	-	5	-	-
		2	855530	31	-	0.0203	ns
		5	4536728	54	-	ns	-2.89; −0.04
		5	8577499	-	11	-	-
		7	4291016	-	14	-	-
		7	16157837	70	-	ns	-3.21; −0.03
		9	7174065	87	-	ns	0.14; 3.81
		9	11190208	-	18	-	-

Non-significant at 5%.

We found one or more candidate genes for each SNP with a significant association at a FDR of 10% and/or a significant frequency change at 0.05% (seeTables S1 andS2 in the Supplementary Material). In general, the identified candidate genes are involved in starch biosynthesis, lipid metabolism, cell wall polysaccharide (hemicellulose, cellulose, and pectin) biosynthesis, and storage protein metabolic/catabolic processes such as α-zein synthesis. Expression levels of these candidate genes in seeds (embryo, endosperm, and pericarp) are variable, generally ranging from intermediate to high depending on the reproductive stage (R1 to R4).

## Discussion

Selection based on expansion volume indirectly led to a decrease in the number of polymorphic SNPs and in the number of SNPs in Hardy–Weinberg equilibrium, and an increase in expected heterozygosity, F_ST_, and D and r^2^ values in populations derived from Viçosa after two or four non-inbred progeny selection. The selection procedures also caused several non-random changes in SNP frequencies. Theoretically, the possible causes are selection (indirectly, due to linkage disequilibrium between the SNPs and QTLs for quality), genetic drift (due to finite population size), migration, and mutation. Migration and mutation should be irrelevant causes. The inclusion of the simulated populations evidenced that genetic drift is not a relevant cause. Notice the equivalence between the parameters estimated in the populations with sample sizes 300 (lower genetic drift) and 100 (higher genetic drift). It should be also highlighted that the average random change in SNP frequencies in the simulated populations was lower than the average changes in the popcorn populations.

Newell *et al.* (2014)observed an increase in LD between SNPs having significant associations with methionine levels over cycles of divergent selection for methionine content. The LD increase occurred for linked and unlinked SNPs. They also observed changes in allele frequencies for two genes controlling methionine concentration. At the*cys2* locus, one allele showed a decrease with selection for high methionine content (from 0.25 to 0.01) and an increase with selection for low methionine content (from 0.25 to 0.74). Wen*et al.* (2011) observed that 57% of SNPs with significant allelic frequency changes among accession regenerations were within flowering-time QTL regions, which was evidence of assortative mating.

Our results revealed greater LD for SNPs separated by more than 10 Mb in tropical populations than in both temperate and simulated populations. In general, tropical populations showed average LD values greater than those of temperate populations. However, LD in the tropical populations was lower than that observed in a secondgeneration composite and higher than that in the composite after 10 generations of random crosses. Truntzler *et al.* (2012) analyzed the extent of LD using a dent maize panel with public and private inbreds. For SNPs separated by 0 to 1,000 bp, the average r^2^ was higher for Syngenta lines (0.61) than for public lines (0.39). For SNPs separated by 1 to 10 Mb, the average r^2^ was 0.03 and 0.04 for public and Syngenta inbreds, respectively. In a study on the extent of LD in commercial maize germplasm, Van Inghelandt*et al.* (2011) observed r^2^ values for unlinked and linked SNPs respectively ranging from 0.009 to 0.013 and 0.020 to 0.029 relative to four heterotic pools.

The differing efficacy of least squares association mapping and Bayesian GWAS to detect true associations, as evidenced by the analysis of the simulated data, can be best attributed to the reduced proportion of phenotypic variance explained by the QTLs. In QTL mapping studies performed by Yongbin*et al.* (2012), Li*et al.* (2006, 2007, 2008, 2009), Babu *et al.* (2006), and Lu*et al.* (2003), the proportion of phenotypic variance explained by QTLs for expansion volume ranged from 3.1% to 35.9%, with average values varying from 4.7% to 15.5%. These high values are due to the phenotyping of progeny or recombinant inbred lines (RILs) instead of plants. In regard to the field data, inefficiency in the identification of QTLs for expansion volume and other quality traits in the breeding populations or in validation of previously identified QTLs can be best explained by reduced heritability. Estimated heritabilities at the plant level for expansion volume in the two temperate populations were 53.2% and 50.7%; these values were lower than the heritabilities at the progeny level observed in the previous QTL mapping studies, which ranged from 72.0 to 83.0% with F_2:3_, BC_1_S_1_, or BC_2_F_2_designs. From an analysis of RILs in four environments, Yongbin *et al.* (2012) mapped seven QTLs for expansion volume and obtained an estimated heritability of 90.0%.

To identify candidate genes for expansion volume and other popcorn quality traits, we based our analysis on kernel physiochemical characteristics affecting expansion volume, such as kernel size, shape, and density as well as kernel moisture, starch, protein, and fatty acid contents. The endosperm is the most important kernel component affecting popping, while starch is the major polymer involved in popcorn expansion. Popcorn kernels contain both vitreous (horny or hard) and opaque (floury or soft) endosperm. During popping, starch granules in the vitreous endosperm are highly expanded and responsible for flake formation, whereas starch granules in the opaque endosperm appear to undergo little change. Acting as a pressure vessel during heating, the pericarp gives popcorn its distinct popping ability. The pericarp is the primary source of fiber in the popcorn kernel, while the germ (embryo) is the primary source of lipids. Other than fracturing the pericarp, popping does not substantially alter either the germ or pericarp. In general, small- to medium kernel size (lower 100-kernel weight) and greater kernel sphericity, kernel density, ratio of vitreous to opaque endosperm, and linoleic acid, oleic acid, and α-zein protein levels are associated with greater expansion volume. Pericarp damage and thickness also greatly affect expansion volume (Sweley*et al.*, 2013).

One candidate gene for SNPs SYN4651 and SYN4646 is GRMZM2G058472 (Gramene ID). The gene product is a glycosyltransferase involved in synthesis of glucuronoxylan, a polysaccharide of the hemicellulose fraction of the cell wall. The gene exhibits intermediate expression level in the pericarp during the middle fruit ripening stage (R3). GRMZM2G060579 is the candidate gene for SNPs PZE-107105783 and SYN36108. This gene encodes an uncharacterized protein involved in pectin biosynthesis and shows an intermediate level of expression in the entire seed (embryo, endosperm, and pericarp) during early to middle stages of fruit ripening (R1 to R4). Tandjung *et al.* (2005) showed that cellulose forms crystalline structures in the popcorn pericarp during microwave heating, thereby improving moisture retention and popping performance, the latter mainly by decreasing the number of unpopped kernels.

The candidate gene for SNP PZE-107083429, GRMZM2G133613, encodes N-acetyllactosaminide 3-alpha-galactosyltransferase. This enzyme participates in glycoprotein synthesis, which is important for endosperm development (Riedell and Miernyk, 1988). The candidate gene for SNPs PZE-110060686, SYN16982, and SYN16979 is GRMZM2G049681. This gene codes for an uncharacterized protein that participates in protein metabolic processes and shows intermediate to high levels of expression in the embryo during early to middle fruit ripening stages. The candidate genes for SNPs PZE-101083826, SYN38509, and SYN38510 are GRMZM2G179521 and GRMZM2G074946; their respective gene products, 6-phosphogluconolactonase and glucose-6-phosphate 1-dehydrogenase, participate in the oxidative pentose phosphate pathway, which is a critical process for maize endosperm starch accumulation (Spielbauer *et al.*, 2013). Surprisingly, these genes show intermediate to low levels of expression in the entire seed during early to middle stages of fruit ripening.

The candidate gene for SNPs PZE-104033459, PZE-104033791, and PZE-104033817 is GRMZM2G138060 (*sugary1*), a determinant of starch composition in maize kernels (James *et al.*, 1995). This gene shows high level of expression in seeds (especially endosperm) during early to middle stages of fruit ripening.

Among the SNPs with significant frequency changes, PZE-104008299, SYN33394, and SYN526 are particularly of interest. Changes in the frequency of these SNPs ranged from 0.23 to 0.38, 0.15 to 0.39, and 0.35 to 0.50, respectively. The SNP PZE-104008299 is located in a region containing at least 12 genes coding for precursors of α-zeins, which are storage proteins accounting for 70% of maize endosperm protein (Holding and Larkins, 2006). All α-zein genes (including 19B1, PMS1, A30, and Z4) are highly expressed in the endosperm during early to middle stages of fruit ripening. The candidate gene for SNP SYN33394 is GRMZM2G429899 (*shrunken-2*), which encodes glucose-1-phosphate adenylyltransferase large subunit 1 involved in starch biosynthesis. Mutation at this locus greatly reduces starch levels in the endosperm (Bhave *et al.*, 1990). The gene is highly expressed in the endosperm during early to middle stages of fruit ripening. Finally, the candidate gene for SNP SYN526 is GRMZM2G007063 (*ohp2*). Similar to the well-known opaque-2 locus (*o2*), this gene also regulates the expression of many members of the zein multigene family of storage proteins (Ciceri *et al.*, 1999). The gene displays intermediate to high levels of expression in the entire seed during early to middle stages of fruit ripening.

To conclude, our results confirm some previously mapped QTLs for popcorn quality traits and provide evidence for several candidate genes affecting starch, storage protein, and oil content of popcorn kernels and pericarp polysaccharide content. The highlighted candidate genes are located in bins 1.04, 3.08, 4.02, 4.05, 5.01, 6.05, 7.02, 7.04, and 10.04. Yongbin *et al.* (2012), Li *et al.* (2006, 2007,2008, 2009), Babu *et al.*(2006), and Lu *et al.*(2003) mapped QTLs for expansion volume in bins 1.04, 3.08, 4.02, 4.05, 5.01, 6.05, 7.03, and 10.04, among others. The main candidate genes affecting starch content are located in bins 1.04, 3.08, and 4.05. Those related to storage protein content are located in bins 4.02, 5.01, and 7.02. Some candidate genes associated with oil content were found in bins 1.01, 3.04, and 7.04. Yanyang *et al.* (2008) mapped QTLs for starch, protein, and oil concentration. Four of the six QTLs for starch content were mapped in bins 1.01, 1.06-1.07, and 4.01-4.02. Three of the seven QTLs for protein content were mapped in bins 4.01-4.02, 7.01, and 7.03. Three of the five QTLs for oil content were mapped in bins 1.03, 3.04, and 7.03.

## Supplementary Material

The following online material is available for this article:

Table S1 - Information on SNPs showing significant association at a false discovery rate of 10% with expansion volume, 100-kernel weight, or kernel density, and on candidate genes.

Table S2 - Information on SNPs with significant allele frequency change at 0.05% and candidate genes.

This material is available as part of the online article from http://www.scielo.org/gmb.
