# Supplementary material for: Linkage disequilibrium, SNP frequency change due to selection, and association mapping in popcorn chromosome regions containing QTLs for quality traits
Source: Genet Mol Biol. 2016 Jan-Mar;39(1):97–110. doi: 10.1590/1678-4685-GMB-2015-0126 (PMC4807383; doi:10.1590/1678-4685-GMB-2015-0126)
Supplement: Supplementary file 1 [file 1415-4757-gmb-39-1-97-Suppl01.pdf]

Table S1 - Information on SNPs showing significant association at a false discovery rate of 10% with expansion volume, 100-kernel weight, or kernel density, and on candidate genes.

SNP	Chr.	Position (pb)	Candidate gene	Position (pb)	Gene product and ontology	Expression
SYN6001	1	2208377	GRMZM2G109725	2205748-2210356	Uncharacterized protein (protein catabolic process)	High level in the seed (endosperm), during mid stage of fruit ripening (R3 to R4)
SYN11901	1	8510819	GRMZM2G009014	8510046-8514842	Uncharacterized protein	Intermediate level in the whole seed (embryo, endosperm (high level in R3), and pericarp), during early to mid stage of fruit ripening (R1 to R4)
PZE-101131103/ PZE-101131166	1	168256682/ 168429768	GRMZM2G018472	168430589-168435266	Uncharacterized protein (protein dimerization activity)	Intermediate to high level in the whole seed, during early to mid stage of fruit ripening
SYN33443	3	15222626	GRMZM2G334628	15220879-15230827	Cell number regulator 8 (carbohydrate metabolic process) (glucose catabolic process)	Intermediate to high level in the whole seed, during early to mid stage of fruit ripening
PZE-104033459	4	41873008	GRMZM2G138060	41396390-41405179	Sugary1 (carbohydrate metabolic process) (maltose metabolic process) (starch biosynthetic process)	High level in the seed (endosperm), during early to mid stage of fruit ripening
SYN4651	6	147905068	GRMZM2G070075	147904224-147915023	Uncharacterized protein	Intermediate level in the whole seed, during early to mid stage of fruit ripening
			GRMZM2G058472	148164946-148170153	Secondary cell wall glycosyltransferase family 8 isoform 1; Secondary cell wall glycosyltransferase family 8 isoform 2 (starch metabolic process) (glucuronoxylan biosynthetic process)	Intermediate level in the pericarp, during mid stage of fruit ripening (R3)
			GRMZM2G121514	147413665-147418505	Beta-N-acetylhexosaminidase (carbohydrate metabolic process)	Intermediate to high level in the embryo, during mid stage of fruit ripening
PZE-107083429	7	125723685	GRMZM2G133613	124733971-124736951	N-acetyllactosaminide 3-alpha-galactosyltransferase (carbohydrate metabolic process) (protein glycosylation)	Intermediate to low level in the endosperm, during mid stage of fruit ripening
PZE-108134983/ SYN20806	8	173931575/ 173973257	GRMZM2G087117	173953988-173958520	Uncharacterized protein	Intermediate to high level in the whole seed, during early to mid stage of fruit ripening
			GRMZM2G002786	174954713-174956656	Seed maturation protein; uncharacterized protein	High level in the seed, during early stage of fruit ripening (R1); high level in the pericarp, during fruit formation (R3)
			GRMZM2G118462	173973120-173978270	Trehalose phosphatase/synthase family protein; uncharacterized protein (carbohydrate metabolic process)	Intermediate level in the whole seed, during early to mid stage of fruit ripening
			GRMZM2G149093	173845613-173848278	Uncharacterized protein (lipid metabolic process) (triglyceride lipase activity) (hydrolase activity)	Intermediate to high level in the embryo, during early to mid stage of fruit ripening

PZE-110012671	10	11184622	GRMZM2G392513	11408729-11421794	Inositol-3-phosphate synthase (carbohydrate metabolic process) (inositol biosynthetic process) (lipid metabolic process) (phospholipid biosynthetic process)	High level in the embryo, during early to mid stage of fruit ripening
PZE-110060686	10	114892724	GRMZM2G049681	114873031-114876662	Putative uncharacterized protein (protein metabolic process)	Intermediate to high level in the embryo, during early to mid stage of fruit ripening
