# Supplementary material for: Linkage disequilibrium, SNP frequency change due to selection, and association mapping in popcorn chromosome regions containing QTLs for quality traits
Source: Genet Mol Biol. 2016 Jan-Mar;39(1):97–110. doi: 10.1590/1678-4685-GMB-2015-0126 (PMC4807383; doi:10.1590/1678-4685-GMB-2015-0126)
Supplement: Supplementary file 2 [file 1415-4757-gmb-39-1-97-Suppl02.pdf]

Table S2 - Information on SNPs with significant allele frequency change at 0.05% and candidate genes.

SNP	Chr.	Position (pb)	Candidate gene	Position (pb)	Gene product and ontology	Expression
SYN6001	1	2208377	GRMZM2G109725	2205748-2210356	Uncharacterized protein (protein catabolic process)	High level in the seed (endosperm), during mid stage of fruit ripening (R3 to R4)
SYN27251/ PUT-163a-5499487-2275	1	2432669/ 2536526	GRMZM2G052546	2535973-2542554	RIP1; uncharacterized protein (carbohydrate metabolic process) (maltose metabolic process) (starch metabolic/catabolic process)	Low level in the whole seed (embryo, endosperm, and pericarp), during early to mid stage of fruit ripening (R1 to R4)
SYN38927	1	2691547	GRMZM2G035377	2655521-2658069	Uncharacterized protein (carbohydrate metabolic process) (glucuronoxylan biosynthetic process)	Non information available
			GRMZM2G388684	2690772-2692141	Xyloglucan:xyloglucosyl transferase (carbohydrate metabolic process) (glucan metabolic process)	Low level in the whole seed, during early to mid stage of fruit ripening
			GRMZM2G085827	2725603-2734211	Holo-[acyl-carrier-protein] synthase (lipid metabolic process) (fatty acid biosynthetic process)	High level in the embryo and intermediate level in the seed (endosperm), during early to mid stage of fruit ripening
PZE-101014266/ PZA-000175002	1	8095694/ 8553473	GRMZM2G099454	7398292-7402945	Chitinase (carbohydrate metabolic process) (chitin catabolic process)	Intermediate level in the seed, during early stage of fruit ripening (R1)
			GRMZM2G009014	8510046-8514842	Uncharacterized protein	Intermediate level in the whole seed (embryo, endosperm (high level in R3), and pericarp), during early to mid stage of fruit ripening
SYN20196	1	15512478	GRMZM2G026807	14623612-14638761	Ribulose-phosphate 3-epimerase (carbohydrate metabolic process) (pentose-phosphate shunt)	Intermediate to low level in the seed (endosperm), during early to mid stage of fruit ripening
PUT-163a-16922676-1073	1	46070067	GRMZM2G058138	46066290-46070815	Uncharacterized protein	Intermediate to high level in the whole seed, during early to mid stage of fruit ripening
			GRMZM5G824405	45641610-45669297	Uncharacterized protein (carbohydrate metabolic process) (fructose metabolic process)	High level in the whole seed, during early to mid stage of fruit ripening
PZE-101083826/ SYN38509/ SYN38510	1	72067065/ 72105795/ 72105859	GRMZM2G179521	72102454-72121802	Glucose-6-phosphate 1-dehydrogenase (carbohydrate metabolic process) (glucose metabolic process)	Very low level in the whole seed, during early to mid stage of fruit ripening
			GRMZM2G074946	73254414-	6-phosphogluconolactonase (carbohydrate metabolic	Intermediate to low level in the

				73259651	process) (oxidative pentose phosphate pathway)	seed (R1) and embryo (R4)
PZE-101120556/ PZE-101120639/ PZE-101120645	1	148525047/ 148566496/ 148566793	GRMZM2G703769	148584485-148585067	Uncharacterized protein	Intermediate to high level in the whole seed, during early to mid stage of fruit ripening
PZE-101131103/ PZE-101131114/ PZE-101131166	1	168256682/ 168257319/ 168429768	GRMZM2G018472	168430589-168435266	Uncharacterized protein (protein dimerization activity)	Intermediate to high level in the whole seed, during early to mid stage of fruit ripening
SYN422	1	236291257	GRMZM2G001606	236290320-236292369	Uncharacterized protein	Intermediate to high level in the seed (endosperm), during early to mid stage of fruit ripening (R2 to R4)
SYNGENTA11666/ PZE-102158721	2	205583015/ 206039441	GRMZM2G408875	205410821-205412632	Phosphoenolpyruvate carboxykinase (ATP) (carbohydrate metabolic process) (gluconeogenesis)	Intermediate to high level in the whole seed, during early to mid stage of fruit ripening
			GRMZM2G142932	205425915-205430161	Uncharacterized protein (protein dimerization activity)	Intermediate to high level in the whole seed, during mid stage of fruit ripening
			GRMZM2G166337	205695705-205697501	R2R3MYB-domain protein (regulation of raffinose metabolic process)	Intermediate to high level in the whole seed, during early to mid stage of fruit ripening
			GRMZM2G367267	205316230-205319784	Cellulose synthase (UDP-forming) (carbohydrate metabolic process) (cellulose biosynthetic process)	Intermediate to low level in the whole seed, during early to mid stage of fruit ripening
PUT-163a-148951348-515/ PZE-103010658	3	5789819/ 5854416	GRMZM2G121452	6304666-6313009	26S proteasome non-ATPase regulatory subunit 8; uncharacterized protein (cotyledon development)	Intermediate to high level in the whole seed, during early to mid stage of fruit ripening
SYN33444/ SYN33443	3	15137742/ 15222626	GRMZM2G334628	15220879-15230827	Cell number regulator 8 (carbohydrate metabolic process) (glucose catabolic process)	Intermediate to high level in the whole seed, during early to mid stage of fruit ripening
			GRMZM5G862219	15048628-15056529	Acyl-CoA dehydrogenase (lipid metabolic process) (fatty acid beta-oxidation)	Intermediate to high level in the embryo and pericarp, during early to mid stage of fruit ripening
PZE-103160210	3	211405876	GRMZM2G125044	211402713-211410775	Uncharacterized protein	Intermediate to high level in the whole seed, during early to mid stage of fruit ripening
			GRMZM2G055585	211614266-211618366	Triacylglycerol lipase (carbohydrate metabolic process)	Intermediate level in the seed, during early stage of fruit ripening (R1)
SYN33394/ PZE-103165953	3	215456783/ 215462316	GRMZM2G048049	215503227-215505485	Expressed protein	Intermediate to high level in the whole seed, during early to mid

						stage of fruit ripening
			GRMZM2G057942	215671743-215676285	Uncharacterized protein (carbohydrate metabolic process) (glucuronoxylan/xylan metabolic process)	Intermediate to high level in the whole seed, during early to mid stage of fruit ripening
			GRMZM2G037431	216010535-216013992	Polygalacturonase (carbohydrate metabolic process) (homogalacturonan degradation)	High level in the embryo, during early to mid stage of fruit ripening
			GRMZM2G429899 (shrunken-2)	216495981-216505345	Glucose-1-phosphate adenylyltransferase large subunit 1, chloroplastic/amyloplastic (carbohydrate metabolic process) (starch biosynthetic process)	High level in the endosperm during early to mid stage of fruit ripening
PZE-104008299	4	5595386	GRMZM2G059620	5548772-5561370	Zein-alpha 19B1 (nutrient reservoir activity)	High level in the seed (endosperm), during mid stage of fruit ripening
			GRMZM2G053120	5544525-5545663	Zein-alpha PMS1 (nutrient reservoir activity)	High level in the seed (endosperm), during mid stage of fruit ripening
			GRMZM2G353272	5539880-5541152	Zein-alpha PMS1 (nutrient reservoir activity)	High level in the seed (endosperm), during mid stage of fruit ripening
			GRMZM2G353268	5535743-5536690	Zein-alpha A30 (nutrient reservoir activity)	High level in the seed (endosperm), during mid stage of fruit ripening
			GRMZM2G404459	5521259-5523302	Zein-alpha Z4 (nutrient reservoir activity)	High level in the seed (endosperm), during mid stage of fruit ripening
PZE-104033791/ PZE-104033817	4	42284265/ 42288134	GRMZM2G138060	41396390-41405179	Sugary1 (carbohydrate metabolic process) (maltose metabolic process) (starch biosynthetic process)	High level in the seed (endosperm), during early to mid stage of fruit ripening
PZE-104080384	4	154716758	GRMZM2G004131	154074091-154075878	Farnesyltranstransferase (lipid metabolic process) (isoprenoid biosynthetic process)	Intermediate level in the whole seed, during early to mid stage of fruit ripening
SYN526	5	4254700	GRMZM2G007063	4253958-4257719	opaque2 heterodimerizing protein2	Intermediate to high level in the whole seed, during early to mid stage of fruit ripening
			GRMZM2G099630	4351432-4366692	Uncharacterized protein (carbohydrate metabolic process) (protein glycosylation/N-linked glycosylation) (glucuronoxylan metabolic process) (xylan biosynthetic process)	High level in the whole seed, during early to mid stage of fruit ripening
SYN4651/SYN4646	6	147905068/ 147909387	GRMZM2G070075	147904224-147915023	Uncharacterized protein	Intermediate level in the whole seed, during early to mid stage of fruit ripening

			GRMZM2G058472	148164946-148170153	Secondary cell wall glycosyltransferase family 8 isoform 1; Secondary cell wall glycosyltransferase family 8 isoform 2; Uncharacterized protein (starch metabolic process) (glucuronoxylan biosynthetic process)	Intermediate level in the pericarp, during mid stage of fruit ripening (R3)
			GRMZM2G121514	147413665-147418505	Beta-N-acetylhexosaminidase (carbohydrate metabolic process)	Intermediate to high level in the embryo, during mid stage of fruit ripening
PZE-106100720	6	153502756	GRMZM2G389089	152682292-152683790	Protein-synthesizing GTPase (lipid metabolic process)	Intermediate to high level in the whole seed, during early to mid stage of fruit ripening
PZE-106115889/ PZE-106116148/ PZE-106116156	6	161854167/ 161924948/ 161990361	GRMZM2G164676	161750718-161815082	Beta-galactosidase (carbohydrate metabolic process) (glucuronoxylan metabolic process) (xylan biosynthetic process)	Intermediate level in the seed, during early stage of fruit ripening (R1)
SYN12692/ PZA02688.2/ SYN12698	6	164180276/ 164183687/ 164186726	GRMZM2G085483	164074969-164076264	Uncharacterized protein (carbohydrate metabolic process) (pentose-phosphate shunt)	High level in the embryo and endosperm, during early stage of fruit ripening
			GRMZM2G085641	164063630-164068010	Uncharacterized protein (lipid metabolic process)	Intermediate level in the embryo, during mid stage of fruit ripening (R4)
PZE-107083429	7	125723685	GRMZM2G133613	124733971-124736951	N-acetyllactosaminide 3-alpha-galactosyltransferase (carbohydrate metabolic process) (protein glycosylation)	Intermediate to low level in the endosperm, during mid stage of fruit ripening
PZE-107105783/ SYN36108	7	157912189/ 157914433	GRMZM2G000453	157913771-157914605	Expressed protein (lipid metabolic process)	Intermediate level in the seed, during early stage of fruit ripening (R1)
			GRMZM2G008309	157718341-157719866	Fiber expressed protein	High level in the embryo, during early o mid stage of fruit ripening
			GRMZM2G060579	158336653-158342207	Uncharacterized protein (carbohydrate metabolic process) (pectin biosynthetic process)	Intermediate level in the whole seed, during early to mid stage of fruit ripening
PZE-107105855	7	157934803	GRMZM2G000453	157913771-157914605	Expressed protein (lipid metabolic process)	Intermediate to low level in the whole, during early to mid stage of fruit ripening
PZE-108004908	8	5025189	GRMZM2G017555	5330395-5332835	Pectinesterase (pectin catabolic process) (carbohydrate metabolic process)	Intermediate level in the whole seed, during early to mid stage of fruit ripening
			GRMZM2G416836	5488159-5500321	Putative trehalose phosphatase/synthase family protein (carbohydrate metabolic process) (trehalose biosynthetic process)	Intermediate level in the seed (endosperm and pericarp), during early to mid stage of fruit ripening
			GRMZM2G029731	4616570-	Inositol-1-monophosphatase (carbohydrate metabolic	Intermediate to high level in the

				4620398	process) (inositol biosynthetic process)	whole seed, during early to mid stage of fruit ripening
PZE-108052599/ PZE-108052600/ PZE-108052603	8	92730672/ 92730702/ 92732462	GRMZM2G022768	91588208-91592778	N-acetyllactosaminide 3-alpha-galactosyltransferase (carbohydrate metabolic process) (protein glycosylation)	High level in the seed (endosperm), during early stage of fruit ripening
PZE-108134983/ SYN20808/ PZE-108135203	8	173931575/ 173953651/ 174257043	GRMZM2G087117	173953988-173958520	Uncharacterized protein	Intermediate to high level in the whole seed, during early to mid stage of fruit ripening
			GRMZM2G002786	174954713-174956656	Seed maturation protein; uncharacterized protein	High level in the seed, during early stage of fruit ripening (R1); high level in the pericarp, during fruit formation (R3)
			GRMZM2G118462	173973120-173978270	Trehalose phosphatase/synthase family protein; uncharacterized protein (carbohydrate metabolic process)	Intermediate level in the whole seed, during early to mid stage of fruit ripening
			GRMZM2G149093	173845613-173848278	Uncharacterized protein (lipid metabolic process) (triglyceride lipase activity) (hydrolase activity)	Intermediate to high level in the embryo, during early to mid stage of fruit ripening
SYN17231/ PZE-110006423	10	4990421/ 4993061	GRMZM2G004534	4697235-4702142	Pyruvate kinase (carbohydrate metabolic process) (glycolytic process)	Intermediate level in the seed (embryo and pericarp), during early stage of fruit ripening
			GRMZM2G003732	4641206-4667439	Uncharacterized protein (embryo development)	Intermediate to high level in the whole seed, during early to mid stage of fruit ripening
PZE-110007091/ PZE-110007194/ SYN16757	10	5485252/ 5537112/ 5592617	GRMZM2G051637	5591911-5595821	Putative receptor protein kinase CRINKLY4 (lipid storage) (cotyledon development)	Intermediate to high level in the whole seed, during early to mid stage of fruit ripening
PZE-110012640/ PZE-110012671	10	11088851/ 11184622	GRMZM2G392513	11408729-11421794	Inositol-3-phosphate synthase (carbohydrate metabolic process) (inositol biosynthetic process) (lipid metabolic process) (phospholipid biosynthetic process)	High level in the embryo, during early to mid stage of fruit ripening
PZE-110045755/ SYN37480	10	86439624/ 86528026	GRMZM2G004131	87096816-87098996	Farnesyltranstransferase (lipid metabolic process) (triglyceride biosynthetic process)	Intermediate to low level in the whole seed, during early to mid stage of fruit ripening
			GRMZM2G123029	87103468-87106603	Pepsin A (lipid metabolic process)	Intermediate level in the seed, during early to mid stage of fruit ripening
SYN16982/ SYN16979/ PZE-110060686	10	114875299/ 114876217/ 114892724	GRMZM2G049681	114873031-114876662	Putative uncharacterized protein (protein metabolic process)	High level in the embryo, during early stage of fruit ripening
